# 3D indoor modeling and game theory based navigation for pre and post COVID-19 situation

**DOI:** 10.3389/fpubh.2023.1301607

**Published:** 2023-11-27

**Authors:** Jaiteg Singh, Noopur Tyagi, Saravjeet Singh, Babar Shah, Farman Ali, Ahmad Ali AlZubi, Abdulrhman Alkhanifer

**Affiliations:** ^1^Chitkara University Institute of Engineering and Technology, Chitkara University, Punjab, India; ^2^College of Technological Innovation, Zayed University, Dubai, United Arab Emirates; ^3^Department of Computer Science and Engineering, School of Convergence, College of Computing and Informatics, Sungkyunkwan University, Seoul, Republic of Korea; ^4^Department of Computer Science, Community College, King Saud University, Riyadh, Saudi Arabia; ^5^Department of Computer Science, King Saud University, Riyadh, Saudi Arabia

**Keywords:** route planning, COVID-19, pandemic, game theory, simulation, localization

## Abstract

The COVID-19 pandemic has greatly affected human behavior, creating a need for individuals to be more cautious about health and safety protocols. People are becoming more aware of their surroundings and the importance of minimizing the risk of exposure to potential sources of infection. This shift in mindset is particularly important in indoor environments, especially hospitals, where there is a greater risk of virus transmission. The implementation of route planning in these areas, aimed at minimizing interaction and exposure, is crucial for positively influencing individual behavior. Accurate maps of buildings help provide location-based services, prepare for emergencies, and manage infrastructural facilities. There aren’t any maps available for most installations, and there are no proven techniques to categorize features within indoor areas to provide location-based services. During a pandemic like COVID-19, the direct connection between the masses is one of the significant preventive steps. Hospitals are the main stakeholders in managing such situations. This study presents a novel method to create an adaptive 3D model of an indoor space to be used for localization and routing purposes. The proposed method infuses LiDAR-based data-driven methodology with a Quantum Geographic Information System (QGIS) model-driven process using game theory. The game theory determines the object localization and optimal path for COVID-19 patients in a real-time scenario using Nash equilibrium. Using the proposed method, comprehensive simulations and model experiments were done using QGIS to identify an optimized route. Dijkstra algorithm is used to determine the path assessment score after obtaining several path plans using dynamic programming. Additionally, Game theory generates path ordering based on the custom scenarios and user preference in the input path. In comparison to other approaches, the suggested way can minimize time and avoid congestion. It is demonstrated that the suggested technique satisfies the actual technical requirements in real-time. As we look forward to the post-COVID era, the tactics and insights gained during the pandemic hold significant value. The techniques used to improve indoor navigation and reduce interpersonal contact within healthcare facilities can be applied to maintain a continued emphasis on safety, hygiene, and effective space management in the long term. The use of three-dimensional (3D) modeling and optimization methodologies in the long-term planning and design of indoor spaces promotes resilience and flexibility, encouraging the adoption of sustainable and safe practices that extend beyond the current pandemic.

## 1 Introduction

The pandemic has changed behavior, increasing awareness of health measures (1). Hospitals need to plan routes strategically to reduce exposure and influence behavior. Global Navigation Satellite System (GNSS) and digital maps are indispensable for any navigation system. Digital mapping is collecting, compiling, and formatting geographic data into a virtual image. Primarily, this technology generates accurate representations of a particular area, including major roads and other points of interest within that area. The technology also allows the calculation of distances from one place to another. Most often, digital mapping is done for open geographic areas and is restricted to a demarcation of boundaries of roads, buildings, and other areas of interest only. Such information has proven to be fundamental for any offline navigation system. This could be why numerous studies have only discussed the deployment and optimization of challenges associated with outdoor navigation. Limited literature has promulgated the issues related to indoor navigation. A recent survey indicates that humans spend more than 90% of their time within indoor environments like homes, offices, malls, and colleges (2). These days, more than 150 companies are working on indoor positioning, indoor mapping, tracking, and navigation (3, 4). One such functionality was recently in demand within the healthcare industry during the COVID-19 outbreak (5).

Many patients who tested positive for COVID-19 required hospitalization, and hospitals had to deal with a heavy patient load. This contagious disease made enclosed spaces like hospitals more vulnerable for people working there. Subsequently, it was realized that indoor navigation systems (INSs) could help improve healthcare services and curtail the spread of infectious diseases like COVID-19. Direct contact or interaction with the crowd is the prime reason for the spread of COVID-19. Hospitals and COVID-19 isolation wards needed a viable solution to manage the crowd movement while ensuring minimal contact with COVID-19 patients or caregivers (6). In the last decade, indoor navigation has become a growing topic of choice for researchers. Mapping and modeling of indoor spaces are done to manage and monitor the infrastructure through location-based services. With the global expansion, approx. 95% of urban areas embrace visualizing smart cities, buildings, and infrastructure in three dimensions (3D) (7). Visualization based on 3D maps has recently been proven more effective than 2D maps for navigation and decision-making. The use of three-dimensional (3D) models is highly advantageous in representing spatial data, as it offers a more interactive and realistic visualization. 3D models have the potential to improve understanding of the relationships between different characteristics and their geographical layout. Visualization involves the deployment of multiple technologies, such as the Internet of Things (IoT), Building Information Model (BIM), and QGIS (8–10). QGIS is a widely used open-source Geographic Information System (GIS) that provides a comprehensive set of advanced tools for mapping, analyzing and generating spatial data. QGIS is a spatial modeling tool due to its extensive capabilities in geographic analysis, data visualization, and 3D modeling. The software’s open-source nature and support from its community make it a versatile and cost-effective alternative, which aligns with the financial considerations of the research project (11). Additionally, the use of various plugins and capabilities within the QGIS software played a crucial role in developing intricate and comprehensive three-dimensional representations of the hospital structure and its surrounding environment. In the purview of the previously stated arguments, this manuscript emphasizes the following scientific contributions:

- To explore the feasibility of using 3D maps for indoor management of crowds.
- To propose a spatial distribution model for indoor environments using game theory and strategizing path optimizations.
- To evaluate the performance of the proposed model through case analysis.

Indoor location prediction and localization involve identifying the precise position or coordinates of an object, person, or device within a limited indoor area such as a building, floor, or room. Several technologies and methodologies, including Wi-Fi positioning, Bluetooth beacons, UltraWideBand, and Inertial Measurement Unit (IMU), can be used to achieve this objective. Location prediction requires projecting the future position or trajectory of an object or entity inside an indoor environment by utilizing past data, trends, or algorithms. However, determining the position inside indoor spaces can be challenging due to the complex and ever-changing characteristics of indoor settings.

In healthcare facilities, implementing location prediction and localization techniques can optimize indoor navigation for patients diagnosed with COVID-19. These strategies can effectively contribute to providing optimized routes and directions within the hospital premises. For instance, predicting the possible whereabouts of a patient within a facility can enhance the efficiency of healthcare personnel in navigating the premises, thereby reducing the risk of exposure. For case analysis, this study investigates the feasibility of deploying the proposed model within a medical facility offering treatment to COVID-19 patients. It intends to suggest optimized routes for the transit of COVID-19 patients and caregivers to ensure minimal contact with other patients and visitors.

The rest of this paper is structured as follows. After the introduction section, section 2 details the literature studies of Indoor 3D modeling, Indoor routing, localization and routing in COVID-19-related indoor premises. The methodology of creating a 3D model of a building using a 2D map and route optimization is described in Section 3. A case study using a hospital building for the proposed approach is described in Section 4. The result discussion related to the case analysis is summarized in section 4. Section 5 provides the discussion and future scope of the presented research.

## 2 Related work

This section includes the paper published in 2016–2023 related to route optimization within indoor environments. The backtracking technique to adopt the traveling salesman problem for route optimization was presented as a novel method to organize tourist itineraries inside a place (12). Similarly, a dynamic technique that uses real-time situation awareness to optimize indoor evacuation routes was proposed. The escape path is dynamically optimized using information about the changing internal environment and a real-time understanding of fire characteristics (13). In the context of smart buildings, a creative solution that manages visitors within hotels, conference centers, universities, and hospitals during the COVID-19 and other pandemics has been planned (5). A reliable and affordable indoor navigation system was proposed to navigate people inside smart buildings. The primary contribution was a new proximity-based navigation system that used beacon data to determine the user’s location. Further, it finds the ideal route for navigating the edge computing infrastructure and provides access to the user over a smartphone (6). After analyzing the influencing variables, a multi-route choice model based on game theory was proposed for path recommendation (14).

As buildings become more extensive, accurate spatial information is crucial for creating indoor route plans. Building information modeling (BIM) technology, which can provide detailed semantic and geometric information, is required to improve indoor route planning. A 3D Geographic Information System (GIS) environment to create a Historic Building Information Modeling (HBIM) model to provide thorough and practical documentation for multi-scale evaluations of cultural heritage was proposed (15). The community of architectural engineering and construction (AEC) benefits greatly from the creation of 3D building models. However, it is difficult and time-consuming to manually create a polygonal 3D model of a collection of floor plans. Currently, scientists are attempting to automate a 3D building’s model reconstruction (16, 17). The development of an indoor space model initially requires the right implementation of sensors to gather data from the indoor environment, which aids in precisely modeling the entire indoor environment and contributes to the effectiveness of the reconstructed model (11, 18, 19). Different technologies and techniques were used in various models for path optimization and route planning, as summarized in Table 1.

**Table 1:** Route planning technologies for different models.

Reference	Year	Model used	Algorithm	Technologies
Carrera et al. (20)	2018	Stochastic model	Tracking Algorithms	Wireless fidelity (Wifi) received signal strength indicator (RSSI), Smartphones.
Fisac et al. (21)	2019	Non deterministic model	Game theoretic trajectory planning algorithm	Autonomous driving technologies
Dogan et al. (22)	2020	Two Dimensional model	Fuzzy c-means clustering algorithm	Bluetooth based technology
Hassija et al. (23)	2020	Drone charging model	Scheduling algorithm	Hedera hashgraph technology
Alamri (24)	2021	Ontology model	Routing algorithm & density rules algorithm	WiFi, Bluetooth, RFID, semantic web technology
Shin and Moon (25)	2021	YOLOv4 model	DeepSORT, multiple object tracking algorithm	Computer Vision technology
Jia et al. (26)	2022	Neural network model	Fingerprinting	Wifi RSSI

Various technologies such as radio frequency (RF), computer vision, and sensors are used for indoor positioning and navigation, including Wi-Fi, Bluetooth, and ultra-wideband (UWB) (27). The study introduces an indoor positioning system that utilizes STM32 and UWB technology. The system demonstrates a notable accuracy level of 20 cm (28). Similarly, a study presents a wireless-inertial fusion positioning system called SmartFPS, which utilizes a stateful LSTM network and a multi-task learning approach. Field testing has demonstrated that SmartFPS exhibits superior performance compared to filter-based approaches, as it achieves an average placement accuracy of 0.575 meters for various pedestrian scenarios (29). Furthermore, a hybrid method for improving indoor positioning accuracy using Bluetooth Low Energy (BLE) fingerprinting and PDR was proposed. Similarly, the use of a Radio Signal Strength Indicator (RSSI) and Pedestrian Dead Reckoning (PDR) for indoor positioning achieves a 68 cm positioning accuracy through sensor fusion (30). The experiment uses the Kalman Filter as a benchmark, and ANN and SVR are combined to achieve a positioning root-mean-squared error of 212.21 cm (31).

## 3 Methodology

Visualizing the precise location of building rooms and utilities in 3D helps visually communicate indoor spatial information. 3D visualization would decrease routing uncertainty inside the structures and help in more informed navigation strategies. LiDAR is used to generate point clouds. These point clouds representing features in 3D space are produced using Light Detection And Ranging (LiDAR) scanners. Despite the rapid advancement of LiDAR-based technologies to enhance data collection capabilities, there are no standardized procedures for classifying point cloud data to extract features of relevance for indoor environments. A manual process was developed to efficiently classify indoor point clouds using LiDAR technology, with a specific focus on identifying traits that are relevant to public safety personnel (32). A proposed unsupervised segmentation method for large-scale 3D point clouds has the potential to improve object-based classification (33). Moreover, a unique approach for classifying indoor components with few labeled samples was introduced to tackle the challenge of insufficient data for training deep learning algorithms (34). Researchers have acknowledged the absence of standardized protocols for point cloud data classification, particularly for extracting relevant features in indoor settings.

Using Volunteered Geographical Information (VGI) is prominently used for the virtual creation of geographic information (35). The third choice involves digitizing the building information for future usage using professional GIS tools like Quantum GIS (QGIS) (36). QGIS provides geometric representation for spatial analysis and semantic representation of building models with different features. For example, if we give the building details like room, floor, and walls, it can be used for various applications (37). The relational data model used by QGIS makes it simple to represent information from a variety of GIS data formats, including Esri shapefiles and GeoJSON. The mapping of a more intricate data model is still necessary. Additionally, we think that QGIS can transform 3D building models because of its extensive support for GIS formats and the use of the Geospatial Data Abstraction Library (GDAL).

**Figure 1:**
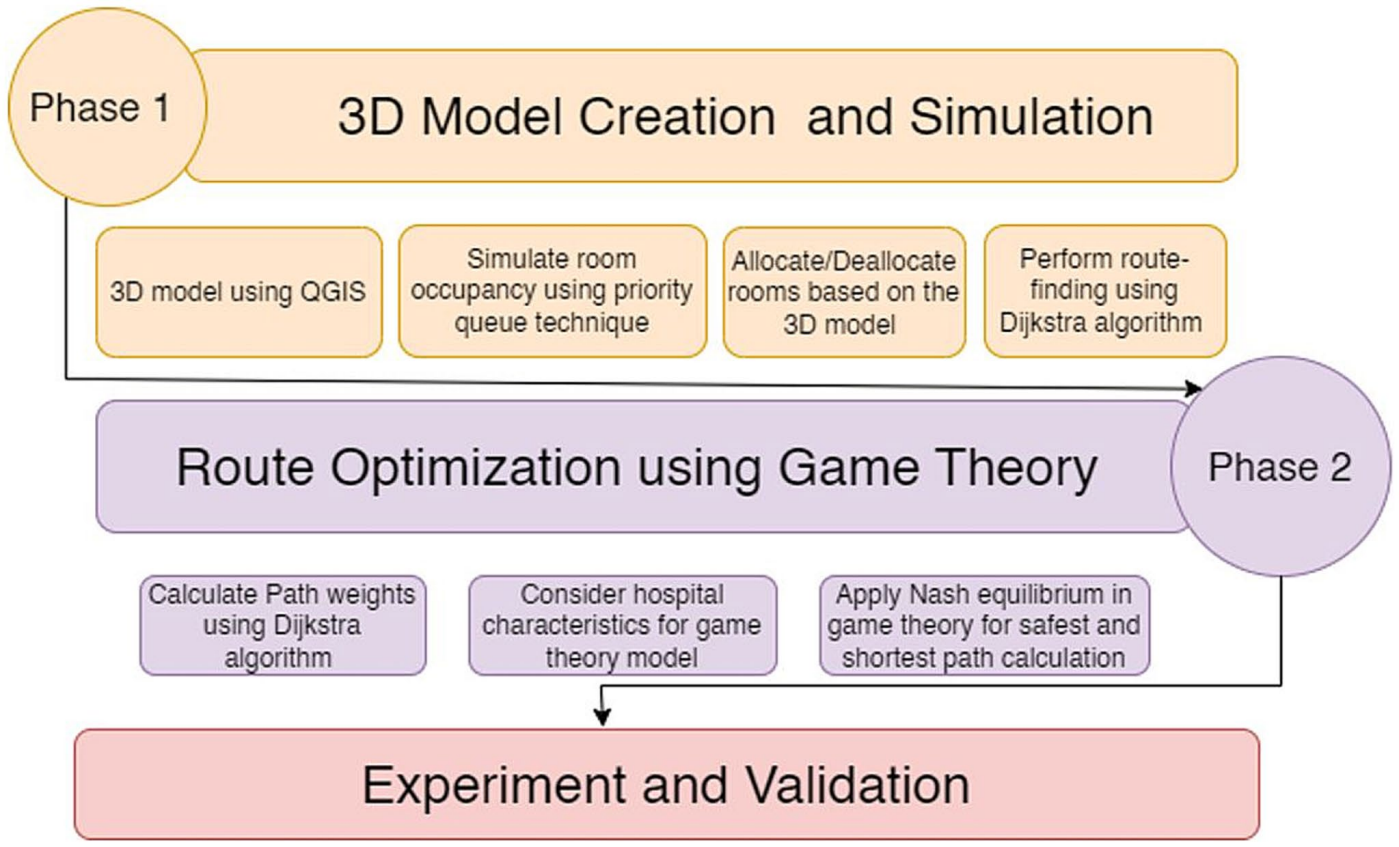
Methodology for 3D indoor modeling and game theory-based navigation.

Game theory is an efficient mathematical theory to address conflict and cooperation. It abstracts all conflict and cooperative phenomena into a game model with three components: participants, strategy, and payout mechanism (38). The key concept is to treat the model-driven and data-driven evaluating techniques as players in a game. The fusion accuracy and variance are regarded as game information, with the weight of the two approaches serving as the game’s weight. The methodology section is divided into four subsections: data collection, spatial data creation, visualization, rendering of 3D information, and route optimization. The study of optimized indoor navigation for COVID-19 patients and hospital resource allocation through a 3D hospital model creation and simulation is shown in Figure 1. The model used a priority queue and Dijkstra algorithm to find the quickest and safest paths. Game theory and Nash equilibrium were used to find the most efficient routes incorporating hospital factors. The methodology was validated through practical trials in a multi-story building, with positive results. This provides a solid foundation for addressing indoor navigation and resource allocation in healthcare facilities, particularly during the COVID-19 pandemic. Facts related to the attributes of a building were accumulated during data collection. These fundamental facts were fetched from OpenStreetMap (39, 40). After including the available 2D information from OSM, a 3D model was created using spatial statistics. The 3D version was visualized and rendered with the usage of plugins. Figure 1 visualizes the methodology of creating a 3D model. When we have a 3D perspective, game theoretic routing methods are used to obtain the ideal path.

### 3.1 Data collection

The following procedure was adopted to collect the spatial information during the experimental setup:

(a) Collect the ground control points by using a LiDAR sensor (Latitude and longitude).
(b) Open layer plugins like Google map, OpenStreetMap, and Bing Map were used to verify the Latitude and Longitude points related to the identified location.
(c) This georeferencing is projected in the decided Coordinate Reference System (CRS), which is supported as the raster layer of the map.
(d) QGIS supports dxf files, shapefiles, map info, PostGIS, and personal geodatabase. Shapefile equipment creates the vector layer over the georeferenced raster layer map.
(e) To express the congestion factor and shorten the computation time, we opt for the most crucial variable, i.e., population density.

All the vector layers are required for digitizing the map. The various features of a building are described in Table 2, along with their mapping method.

**Table 2:** Feature description and mapping method.

QGIS indoors feature classes	Description	Mapping method
Facilities	Building in data collection	Digitize as a polygon in QGIS
Levels	Separate floors of the building	Digitize as a polygon in QGIS
Walls	Separate rooms within one floor	Digitize as line in QGIS
Units	The space is enclosed by a floor and separated by walls	Digitize as a polygon in QGIS
Points of interest	Population density or locations where the path will be decided	Points

### 3.2 Spatial data management

Most high-level data processing procedures are automated in the QGIS. The tools are arranged as distinct toolboxes called geoprocessing tools. Figure 1 shows the tools in the correct order. Creating a SpatiaLite database is the first step in developing the information modeling process that supports the management of indoor GIS data within QGIS. The use of a vector layer allows the creation of new shapefiles.

The input data used to build the topological model of the building might originate from a variety of sources and forms. Vector data, such as Shapefiles (shp.), AutoCAD (dwg), or MicroStation (dgn) files, are frequently used to store data and information in the form of floor plans. Additionally, the relevant data from the BIM model can be exported and used to create floor plans as Shape, Vector, or CAD files. If data related to floor plans is already accessible in picture form, it must first be georeferenced before being manually digitized. The floor map will then be ready after importing vector shape files using QGIS Toolbox. The many indoor feature classes include different geometry types, such as polygons, lines, points, and multipoints. Information like an id, height, name, etc., is contained in these feature classes. Additionally, users of QGIS can import Map Layouts. The user must assess and fix any potential issues after running this operation.

**Figure 2:**
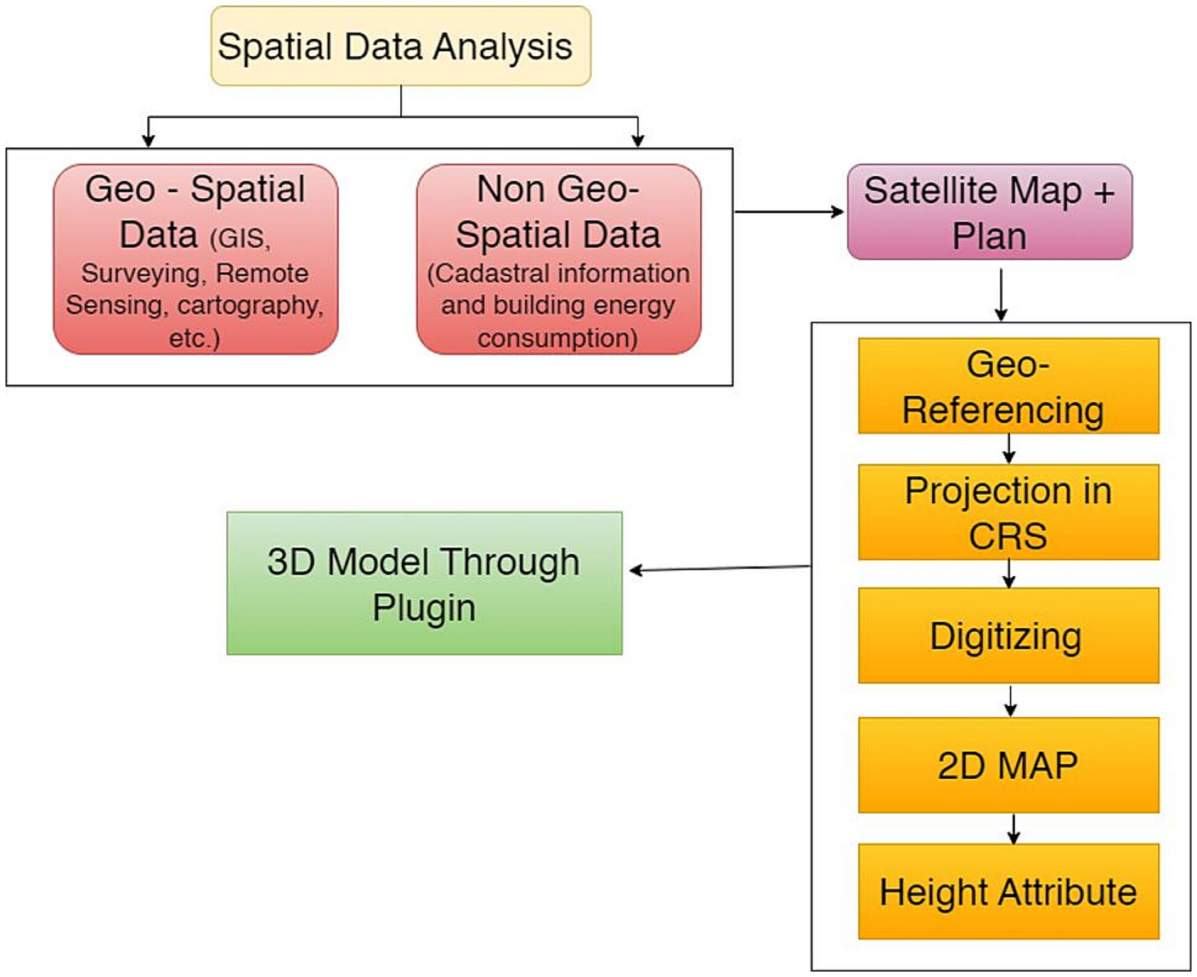
Conceptual chart for generating a 3D model from a 2D map using the GIS platform.

The final step is to create a 3D model by using the collected information. To visualize the model in three dimensions, one can incorporate the altitude/height data into the project by double-clicking the layer properties. Each layer has features associated with it, such as id, height, name, etc. The altitude attribute can show the desired altitude by using Equation 1. A 3D model can be generated using a 2D map, as shown in Figure 2.


(1) Expression=feature∗n

Where the feature is id and *n* is the number of height.

#### 3.2.1 Visualization and rendering of 3D model

This component is used for determining the precise location and heights of a building or any other point features within the building. LiDAR and conventional methods (such as tape measures, theodolites, total stations, and leveling instruments) were employed to gather information on the building’s feature points. Additionally, Architectural drawings and blueprints, if accessible, may be utilized as a component of the conventional data-gathering process. The probable topological errors that can affect the visualization of the model are detected and eliminated. After self-inspection of collected data, duplicate data are removed. Then, a LiDAR sensor is used to estimate each point’s height within the shapefile. The QGIS plugin “qgis2three.js” was subsequently used to create a 3D model of an entire building.

#### 3.2.2 Route optimization based on game theory

Many elements may impact the path selection criteria within a data model. Game theory is used to select the path by using a path-benefit parameter. This parameter is primary to optimize the data model. This value is then provided as an input parameter for the game theory to select the optimal path. Game theory is a particularly effective mathematical framework for defining and addressing path-planning issues (41). The use of game theory allows us to create models that capture the complex decision-making processes of various stakeholders in the medical environment. It also helps us optimize the allocation of resources, such as unoccupied rooms and the most efficient routes. The Nash equilibrium concept in game theory is particularly useful for strategic path planning, finding the right balance between safety, efficiency, and patient preferences. Finally, it promotes fairness and effectiveness in allocating resources and navigation techniques, which are crucial for a successful indoor navigation system in hospitals.

It examines the interactions between two or more rational agents called players. In these situations, other players, known as the environment, are typically added to the game to represent the uncertainty that influences the players’ decisions. A wide range of factors influences the decision of which strategy to use. The most crucial is the player’s utility function, which measures how satisfied the player is with each potential strategy profile. Since the outcome of a game also depends on the decisions made by the other players, in most situations, using a strategy that enables a player to maximize utility may result in a noticeably lesser payoff. To put it another way, it is typically straightforward for other players to retaliate against the greedy strategy. This fact has prompted numerous definitions of strategy optimality and logical player conduct. The Nash equilibrium is perhaps the term most frequently used in this context. It refers to any strategy profile from which one player cannot benefit from a unilateral deviation. In this experiment, multiple entry and exit doors are available to the patient. The patient looking for the room acts as a player, and the natural parameters are room location, patient condition, hospital crowd information, and available routes. Mathematically, a Nash solution is a pair of decisions a*, b* that satisfy the inequalities in a game with two users. Here, a and b symbolize the decision variables of users 1 and 2, respectively. At the same time, U1(a, b) and U2(a, b) denote the utilities that these users desire to maximize, as shown in Equations 2, 3 (42).


(2) U1(a∗,b∗)>=U1(a,b∗)foralla∈A

(3) U1(a∗,b∗)>=U1(a∗,b)forallb∈B

where A and B are the corresponding decision spaces for the two users. The concept of the Nash solution is appealing because it results in an equilibrium condition that ensures no user can gain by unilaterally departing from its Nash decision (42). The mapping of relationships between all routes is the Nash equilibrium that the game theory model derives, making it possible to identify the best path for patients.

The optimum path may not always be the shortest since different network components move at different speeds. As a result, the problem of selecting the optimal route in an emergency would be to select the quickest and safest route to the destinations while accounting for walking speed on horizontal indoor floors, vertical walking speed on stairs, and vertical elevator walking speed. We have predicated the cost of each edge based on the time required to cross that edge, which can be calculated using the Equation 4 below (43):


(4) $$\begin{array}{c} Timetotraverse\\ theedge \end{array}=\begin{array}{c} Distanceof\frac{edge}{speed}of\\ traversingtheedge \end{array}$$

The time to cross the edge equals the edge’s length times its speed.

The fastest route between any two places is determined using the Dijkstra algorithm and the aforementioned edge costs. Therefore, using the following calculation (Equation 5), the path with the shortest overall traversing time is the fastest way to get from A to B (43).


(5) $$T=\min\overset{n}{\underset{i£1}{\sum }}\left[\left(\frac{D_{h}}{S_{w}}\right)+\left(\frac{D_{v}}{S_{Stair}}\right)+\left(\frac{D_{path}}{S_{path}}\right)\right]route$$

Where T is a total traversing path, _Dh_ and D_v_ are the Distance walked Horizontally and Distance walked vertically, Dpath is the Distance traversed on the path, S_w_ is the speed of horizontal walking, S_stair_ is the speed of vertical walking, S_path_ is the speed on the path, and L is the total Distance traveled (L = D_(vertical)_ + D_(horizontal)_ + D _(path)_).

The navigation path between two desired destinations can be determined using Dijkstra based on dynamic programming, and the fastest path can be found. Dynamic programming can be used to solve the node relation graph in the best possible way. The patient can choose their commute by looking for the shortest Distance or the fewest nodes. The infection rate impacts COVID patients’ movement (Intensive, moderate and normal). The average pace of a patient using crutches ranges from 0.63 to 1.35 meters per second (m/s), walking sticks from 0.26 to 1.60 m/s, an electric wheelchair from 0.85 to 0.93 m/s, and a manual chair or stretcher from 0.13 to 1.35 m/s (44). The patient’s evacuation time may be impacted by the number of individuals, their density, their movement pace, and the breadth of the corridors.

We pairwise compare the two paths when there are several options before selecting the optimal one. According to the generic definition of the game, G = R, S, A, and U, we have the following:

1. Patient: The definition of the ith route is Ri, iN, N = 1, 2… n.
2. R_i_ is S_i_, S_i_ [0, G_i_], I belong to N in the strategy space. Ri leads us to believe that Gi has the greatest number of patients.
3. The payout function of the game is U(x_1_,…, x_n_); to put it more precisely, let.

(6) $$U\left(x_{1},x_{2}\ldots \ldots ..x_{n}\right)=\left\{\overset{n}{\underset{i=1}{\sum }}rs_{i}^{p}*t_{i}+\overset{m}{\underset{j=1}{\sum }}rs_{j}^{l}*t_{j}\right\}\;$$

Where $rs_{i}^{p}$ is the suggested score for the ith path, and $rs_{j}^{l}\;$ is the suggested score for the ith rooms, as mentioned in Equation 6 (44). In addition, n is the number of roads, m is the number of rooms, and t is the amount of time spent traveling or in a room. The Nash equilibrium represents the mapping relationship between all routes that the game theory model derives, making it possible to identify the best course of travel.

## 4 Case study

The geographical area of a multistoried building with multiple entry and exit options at the GPS coordinates 30° 30′ 59.2524” N and 76° 39′ 33.1560″ E has been considered for this experiment. There were three floors in the building. The Building Information Model (BIM) model was created using a spatial database in QGIS after collecting LiDAR data. Elements such as walls, rooms, floors and their respective attributes have been mentioned separately in shape files for each floor. TFmini-s handheld LiDAR sensor with an accuracy of 0.1–12 meters was used to collect data at medium walking speed. The scan time was less than equal to 30 s. These techniques included moving gradually and without abrupt changes in direction, keeping motion to two axes at once, and more. Scanning a large multi-story building of 746.889 m^2^ (Cartesian) took hours, depending on the characteristics of the building.

### 4.1 2D map

Coordinate Reference System, i.e., EPSG: 4326-GS 84 and Latitude and longitude were used to locate the building on Google Maps. After measuring the dimensions of different areas, these points are extracted in the QGIS version 3.24.1. The building block has a different feature in the form of polygon layers. Each polygon has its axis as Latitude, longitude, height, and area. The Latitude and longitude of each Floor come from the georeferenced map.

### 4.2 Visualize 3D data model

The subsequent processing steps were carried out in QGIS software using the Qgis2three.js plugin. Qgis2three.js plugin uses the Three.js Library to visualize the floor map in three dimensions. It is compatible with any web browser that supports WebGL. The following steps were performed to load all data into QGIS:

(1) Creating the two-dimensional model of a building required the development of the floor plans to develop a 3D model of the same.
(2) After successfully merging the floor-to-floor height into the level and creating all the floor plans for all levels, the walls may be added to produce a simple three-dimensional model or block model.
(3) After confirming the height data, the level information is automatically incorporated within the QGIS2threejs.exporter window.
(4) There was a need to add details to the fundamental three-dimensional model, such as opacity expression and color-feature style, to distinguish each feature class included in the QGIS model’s hierarchical structure.
(5) The inner and outer walls were added by choosing the wall option from the layer window. The wall type and dimension can be chosen and altered as desired. A basic 3D model is created after adding all the walls.
(6) After creating the primary 3D model, specific details related to rooms, stairs, reception area, seating area and other components were added.
(7) Introduce the height in each polygon layer in the properties/attribute table by adding the new height column. Then, with the plugin “qgis2threegs,” the 3D model is directly generated.

### 4.3 Route optimization based on game theory

In this study, the optimal path must be determined to find the safest route to transit COVID-19 patients. We evaluated different options before selecting the optimal path. The chosen building has three entrances with a dedicated block assigned to COVID-19 patients.

**Figure 3:**
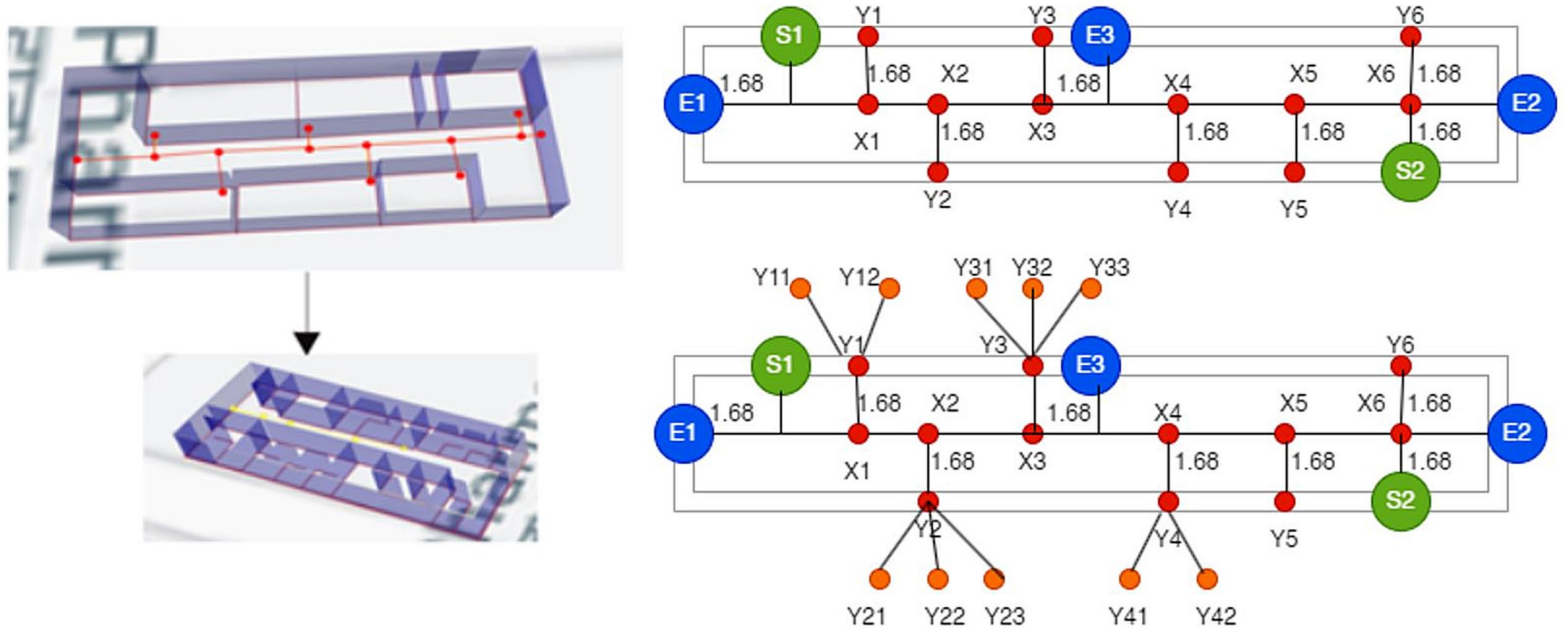
A model of a three-dimensional building with hierarchical relationships.

As shown in Figure 3, a Node Relational Structure (NRS) is proposed to represent the internal architecture of the building. The model represents the facilities as a graph based on the relationships between the nodes and edges to visualize the structure of a room in a building. A simulated model may specifically describe facilities available at a location.

The graph includes nodes N and edges E. The hypergraph of the building is divided into corridors, rooms, bridges, and floors. The network topology created using the NRS model is shown in Figure 3. There are three floors in the building block. Each Level, or F1, F2 and F3, constitutes a separate sub-drawing. A stairway links F1, F2, and F3. In each sub-diagram, a room and a hallway serve as the nodes and edge connections can join the two spaces together. The corridor node is expanded to create an extension node between each room to accurately depict the Distance between the two nodes. Finding the shortest path and safest in this method allows for the precise measurement of the path length. Corridor node is increased to X1, X2, X3, X4, X5, and X6, for instance.

The relationship for connected nodes G = (F1 U F2 U F3), where F1, F2 and F3 are different floors. F = {X U Y U E) where X contains path points, Y contains nodes/rooms/cabins, and E is for entry and exit. F1 = (X1, X2, X3, X4, X5, X6, S1, S2, E1, E2, L1, L2) R_g,_ F2 = (X7, X8, X9, X10, X11, X12, S3, S4, L1, L2) R_f,_ F3 = (X13, X14, X15, X16, X17, X18, S5, S6, L1, L2) R_s,_ R_g_ = (Y1, Y2, Y3, Y4, Y5, Y6), R_f_ = (Y7, Y8, Y9, Y10, Y11, Y12) and R_s_ = (Y13, Y14, Y15, Y16, Y17, Y18). Where G is the union of F1, F2, and F3 floors, and S indicates the stairs. E1, E2 and E3 are entry and exit points for quarantine or recovered patients on the ground floor. L1 and L2 are elevators on the first and second floors. Rooms were further divided into various cabins. The optimized route from node to successor is calculated through a fusion approach, as shown in Table 3.

**Table 3:** Optimal route and associated attributes from node to successor nodes using a fusion approach.

Sr. no.	Node	Immediate precursor	Immediate successor	Optimized route from node to successor
1	X1	E1, S1, L1	X2, Y1	3.735 m
2	X2	X1	X3, Y2	8.557 m
3	X3	X2	X4, Y3	2.177 m
4	X4	X3	X5, Y4	3.628 m
5	X5	X4	X6, Y5	5.180 m
6	X6	X5	E2, Y6, S2, L2	5.770 m
7	Y1	X1	Y11, Y12	1.68 m (from the center of the corridor)
8	Y2	X2	Y21, Y22, Y23	1.68 m (from the center of the corridor)
9	Y3	X3	Y31, Y32, Y33	1.68 m (from the center of the corridor)
10	Y4	X4	Y41, Y42	1.68 m (from the center of the corridor)
11	Y5	X5	-	
12	Y6	X6	-	
13	E1		E2	30.880 m
14	E1		E3	19.560 m

A game theory model was applied to address the issue of repetition. Patients and methods of selection of rooms are included in the model. Incorporating game theory principles into the process of room allocation and deallocation allows for the most efficient use of available rooms, considering factors such as patient requirements, current room occupancy, and other pertinent situations within the hospital setting. This approach facilitates efficient queue management, reduces interpersonal encounters, and promotes adherence to social distancing protocols within the hospital premises, ultimately leading to a safer navigation experience for patients. Game theory models interactions between patients, hospital staff, and administrators to determine the best course of action that ensures safety and efficiency. By creating a game where participants decide the best routes and allocate rooms, aiming to identify the most secure and efficient path for patients while optimizing the distribution of resources.

**Figure 4:**
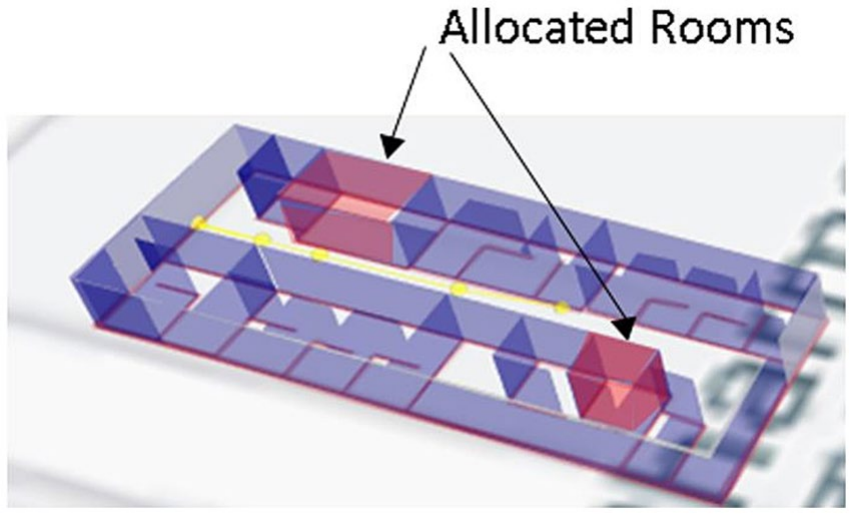
Allocation of rooms.

First, each room is considered a node (V); to create a weighted graph, each edge (E) is assigned the measured Distance in meters. We build a weighted network in which each edge’s weight equals the Distance between any two nodes. The priority queue (PQ) is used to determine the shortest path after implementing the adjacency of the relevant matrix using this network. A PQ algorithm is utilized to assign and release rooms from COVID-19 patients while attempting to minimize patient contact while crossing hallways, as shown in Figure 4. The vacant room having the shortest path is given to the patient at preference. When a COVID-19 patient is healed, they can leave the room with little to no contact with other patients. The steps of allocation and deallocation are mentioned in Table 4.

**Table 4:** Algorithm for room allocation and deallocation based on route algorithm.

Algorithm (LR, C): Where C is the total number of available rooms, and LR is the list of available rooms. N is the total number of patients, and R_f_ is the rooms on the first Floor. Establish a priority queue PQ with a size equal to the total rooms (NR). PQ is the priority queue.Allocation: PQ.Create(NR) Initialize N:=1 Do the following for every registered patient Yi = Allocate a room to the patient using PQ.Delete() LR = extract(LR,Yi) C = C – 1 If N > Y_f_ Shift patient to f + 1th Floor N=N + 1 Repeat until LR is not emptyDeallocation: N=N-1 LR = add(LR, Yi) PQ. Insert (Yi, H). C=C + 1PQ methodsCreate (NR). Establish a priority queue PQ size equal to the total rooms (NR). Insert (Yi, H). Add Yi room to the priority queue following its minimum Distance from entry. Find closest (T). Take out room from priority queue with minimum Distance from point T. Delete(H): Delete the first node with minimum Distance from entry.

**Figure 5:**
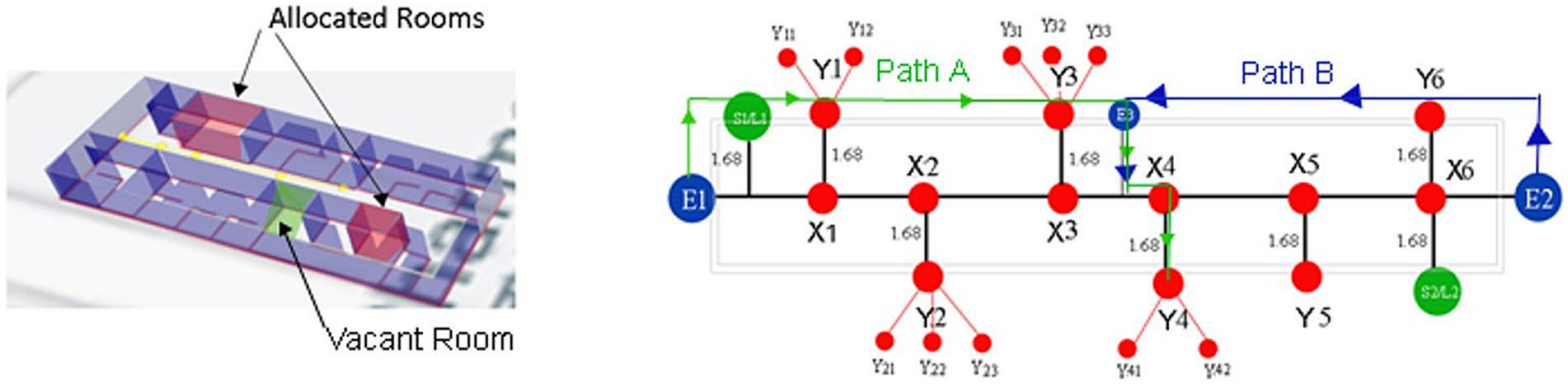
Safest path for a vacant room.

For example, in a route network, COVID patients are given access to some vacant rooms, and separate virtual paths are made by splitting the accessible paths. A parallel route model for COVID patients may consider each virtual path as a route. Patients may be limited to only a certain number of routes (depending on the congestion) between any two nodes in a route by employing room allocation and deallocation information. The path weight is evaluated using the simulation method from QGIS as the selection index for the path selection. Later, in the suggested system, the best path is selected using game theory, and the Nash equilibrium is obtained based on the path weight produced by the fusion approach. Each path is given a weight based on its Distance. Path A is the safest route with the fewest transmission nodes, followed by Path B. Patient-assigned vacant rooms will be determined by priority queue, and patients can select the quickest and safest routes according to Nash equilibrium. Patients are free to choose their path without being aware of other patients’ choices. The proposed strategy can shorten travel times and ease levels of congestion, according to experimental data from the building block, as shown in Figure 5.

**Figure 6:**
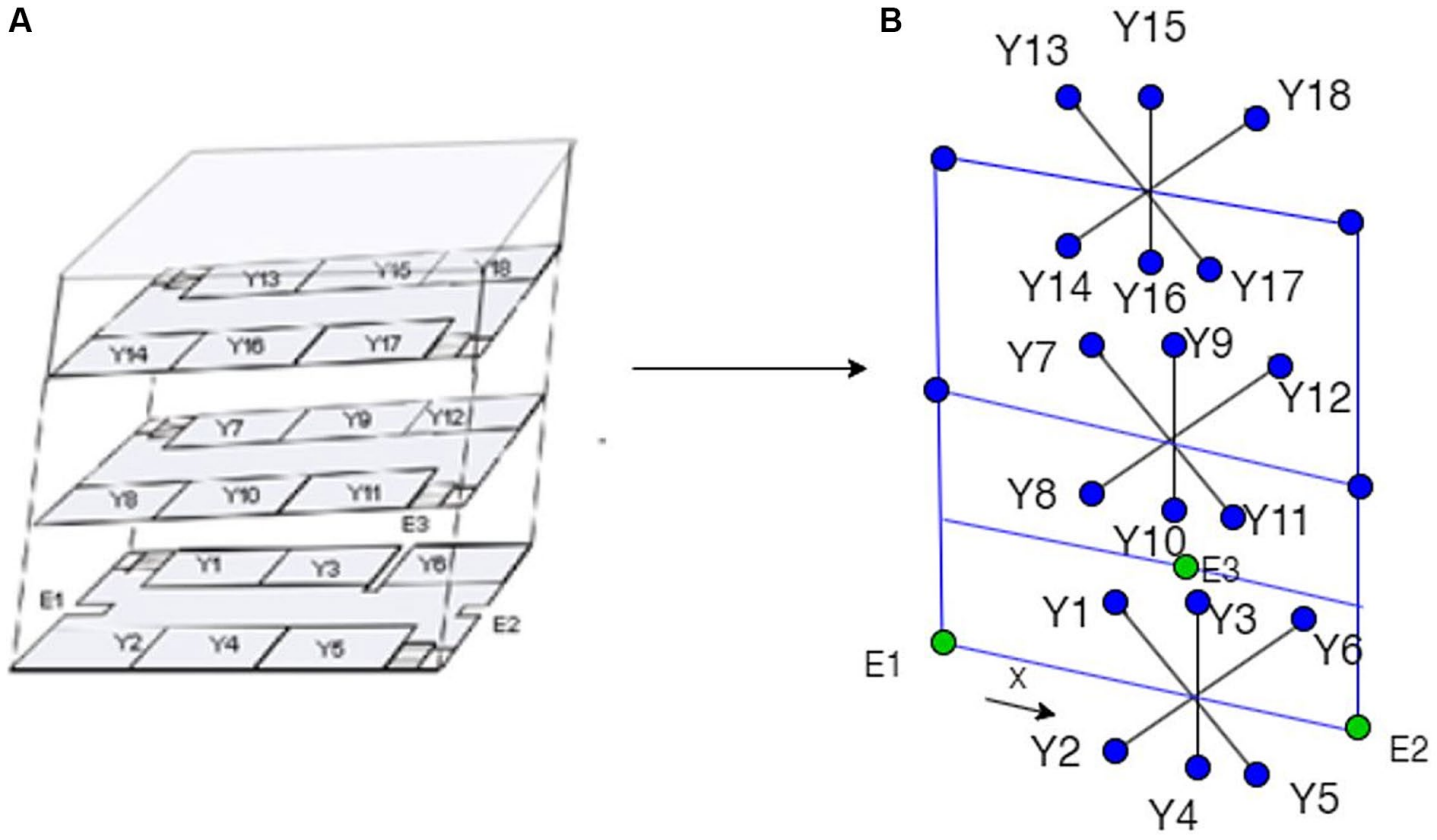
Building the geometric network model **(A)** 3D building model **(B)** topological model.

**Figure 7:**
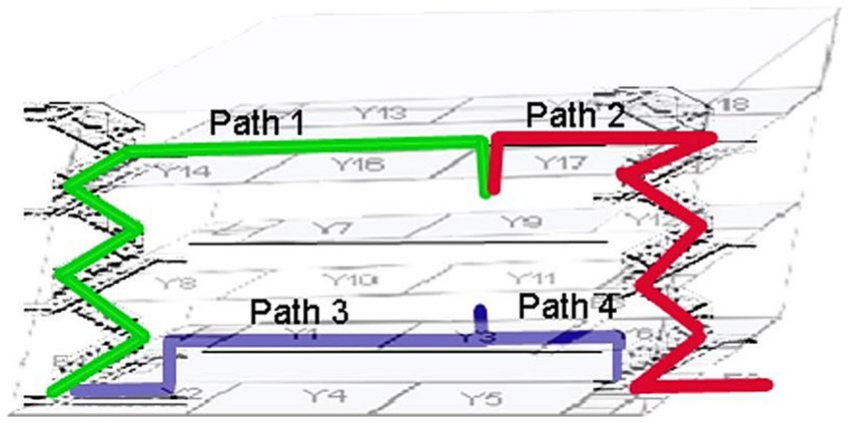
Navigation routes from source to destination.

A geometric network model integrates the topological network model with the geometric data. It would allow for cost computation (based on Distance or optimality) and visualization, as illustrated in Figure 6. Based on a geometric network model where rooms and spaces are connected to corridors, stairs, and elevators connect the levels, the 3D interior network dataset for the sample building was created, as shown in Figure 7.

Another output of the model that is provided based on the level of contamination in various locations and quick routing is the lengths of the shortest, safest short, or optimized safe paths. The formula shown below was defined to calculate the probability of infection (37),


(7) $$n_{v}=\overset{m}{\underset{i=0}{\sum }}\left(\left(\frac{L}{v}\right)*P_{t}\left(i\right)*\gamma_{b}\right).$$

(8) InfectionProbability=nv/Infectionthreshold×100

L is the length of the offered shortest, safest short path, or optimum safe path. V is the average speed at which a person moves. P_t_ (i) represents the amount of contamination at point i. $\;\gamma_{b}$ represent the infection rate.

The Node relation model can be used to determine the best (fastest) routes between any two places inside the building after creating the interior network dataset with the necessary edge costs and connecting the indoor network to the road network (Table 5). This would give emergency personnel a rough idea of the length and Distance needed to get to the desired location inside the structure.

**Table 5:** Navigation path between selected nodes.

Sr. No.	Start	End	Dijkshtra path	Nodes covered from source to destination	Distance
1	E1	Y4	E1->X1->X2->X3->X4->Y4	4	23.512 m (Shortest)
	E1	Y4	E1->E3->X4->Y4	2	33.562 m (Safest)
2	E2	Y4	E2->X6->X5->X4->Y4	3	16.175 m (Shortest)
	E2	Y4	E2->E3->X4->Y4	2	25.882 m (Safest)
3	E1	Y17	E1->S1/L1->Y14->Y16->Y17	2	29.345 m
	E2	Y17	E2->S2/L2->Y17	0	11.456 m (safest and shortest)
	E3	Y17	E3->Y1->Y2->S1/L1->Y14->Y16->Y17	4	29.675 m
	E3	Y17	E3->Y5->S2/L2->Y17	1	19.987 m

Graph theory and Nash equilibrium can be useful to model the behavior of patients in a corridor and to identify strategies that can reduce congestion. However, it is important to note that the effectiveness of these approaches will depend on the accuracy of the model and the ability to implement interventions that are feasible and operative in practice. To address the congestion, some approaches are taken into consideration (45–47):

(1) Identification of the relevant variable and parameters: It includes the number of patients, the physical layout of the corridor, the location of destination, and the time required for patients to travel through the corridor.
(2) Game formulation as a mathematical model: this involves defining the payoff function of a game theory for each patient, which represents their patient’s objective in the game. The payoff function depends upon travel time, the Distance traveled, or other factors related to hospital facilities.
(3) Design interventions to reduce congestion: Based on the results of the game analysis, interventions be used to can reduce congestion. These include changing the physical layout of the corridor, providing signage or other communication tools to guide patient flow, or implementing scheduling policies that spread out patient arrivals and departure.

For better comparison and visualization, we took into consideration a test scenario of a simple storey structure to assess the efficacy of this 3D model in decision-making and improving interior routing times. The task of navigation has been tested on 10 individuals, and the average amount of time it took each of them to go a specific distance from point E2 to point Y17 has been calculated. As a result of this evaluation, a small error or lack of information could result in a significant delay in reaching various points inside buildings. Table 6 shows the average of the computed time and Distance.

**Table 6:** Comparison of proposed model with existing model.

	Without 3D visualization and Dijskhtra algorithm	With 3D visualization and Dijkshtra algorithm using dynamic programming	Time difference
Comparison of Dijkshtra with proposed model	95 s. 32.341 m	72 s. 22.315 m	23 s. 10.026 m

In this study, the data from LiDAR sensors were combined with a simulated model to create a fusion strategy that was based on game theory. There might be differences between established and actual models. Even if there are errors in the developed model, changing the game strategy can still increase the precision of the projected results. The model-driven approach’s biggest drawback is the need to build an extremely accurate model. This could decrease the accuracy of the position estimate findings and potentially cause the algorithm to diverge. This model makes use of the mathematical framework developed in the model-driven technique to suppress the estimation divergence brought on by the measurement error in the data-driven method. The complementing benefits of the two estimation approaches, which operate on various principles, are realized through this study.

Most studies conducted so far focus on objectives, such as network modeling, indoor routing, or visualization goals, making it difficult to draw attention to other crucial data needed for emergency response. There have also been some attempts to use CityGML and Building Information Modeling (BIM) for indoor spatial modeling. However, these models either lack spatial analytical capabilities since they were created for viewing purposes or because they contain extremely complicated architectural engineering elements, as shown in Table 7.

**Table 7:** Relevant studies on 3D models: a comprehensive overview of research findings.

Reference	Year	Database	Software	Tools and technology	Algorithm/Plugins	Output in dimension
Aleksandrov et al. (48)	2019	PostGIS	QGIS	LiDAR	CityGML	3D Outdoor
Liu et al. (8)	2020	PostgresSQL	Revit, AutoCAD, Blender	Python, Java	Map Matching, Path Planning	3D
Ahmad et al. (49)	2020	JSON files	Unity 3D	Mixed Reality Toolkit, Wi-Fi, Augmented reality	the k-nearest neighboring algorithm, machine learning algorithms	3D
Ma et al. (50)	2021	GIS database	AR-GIS	Augmented Reality, BLE, Inertial Sensor	Multi-sensor fusion-based algorithm, particle filter	3D
Trybała and Gattner (51)	2021	ESRI	ArcGIS	Geoprocessing tools, Python	Dijkstra’s, Hill editing and metaheuristic Algorithms	3D
Aziz et al. (52)	2021	Inbuilt database	Phantom 3, Agisoft, Dji GO app	Camera and Unmanned Aerial Vehicles	Inbuilt algorithm of application-	3D
Ours		PostGIS	QGIS	Qgis2threejs, python	Priority queue, Game theory, nash equilibrium, Dijkshtra, dynamic programming	3D

## 5 Discussion

In COVID-19 and post-COVID-19, applying this approach within indoor areas can significantly improve the ability to reach the destination safely. This study uses an innovative indoor navigation system for a hospital building to provide an optimized route for COVID-19 patients. This research provides route optimization in a building by including queue management, intelligent navigation of spaces to minimize human interaction, and social distancing safety mechanisms. The proposed research work was conducted in two phases. In phase 1, QGIS software was used to design the 3D model of a building. The designed model was used as a simulation to examine room occupancy using the priority queue technique. The designed 3D model was further used for the room allocation/ deallocation and route-finding process. For the route-finding process, the Dijkstra algorithm and game theory were used in the second phase of this research. Path weights for the game theory were calculated using the Dijkstra algorithm. In this phase, game theory used the concept of Nash equilibrium to identify the best course of action that is the safest and shortest path. For the safest and shortest path calculation, the game theory model uses hospital characteristics like vacant rooms, Distance, corridors, facilities, staff, people density on the path, and speed of the patient. To validate the design framework, an experiment was conducted on a multi-story building located at GPS coordinates 30° 30′ 59.2524” N and 76° 39′ 33.1560″ E with multiple entry and exit options. The shortest and shortest path for 10 individuals was calculated on eight different routes. According to the performed result analysis, it has been observed that by prioritizing vacant rooms based on patient need and using the most efficient routing algorithm, the simulation optimizes the flow of patients through the hospital, minimizing congestion and wait times. The incorporation of Nash equilibrium ensures that all patients, staff, and administrators have their needs considered, leading to a fair and efficient allocation of resources. This research study also provides the comparison of the proposed framework with other methodologies, and it is observed that overall, this framework has the potential to provide the safest and safest route to the patient by considering the hospital’s current situation and makes it a promising solution for managing hospital facilities in a more effective and sustainable way. The forthcoming research will focus on integrating real-time data and using the Internet of Things (IoT) and machine learning techniques to develop predictive models that allow us to make dynamic adjustments to paths. The research will also incorporate augmented reality to improve navigation. This research will collect user feedback to iteratively improve the design. It will address the challenges of multi-floor navigation and vertical mobility and explore integration with Electronic Health Records (EHR) systems while implementing privacy and security measures. Finally, this research will ensure the scalability and generalizability of the proposed framework.

## Data availability statement

The original contributions presented in the study are included in the article/supplementary material, further inquiries can be directed to the corresponding authors.

## Author contributions

JS: Methodology, Supervision, Writing – original draft, Formal analysis. NT: Conceptualization, Data curation, Investigation, Methodology, Writing – original draft. SS: Conceptualization, Investigation, Methodology, Software, Supervision, Writing – original draft. BS: Validation, Visualization, Writing – review & editing. FA: Formal analysis, Project administration, Supervision, Writing – review & editing. AAA: Funding acquisition, Resources, Writing – review & editing. AA: Funding acquisition, Resources, Writing – review & editing.
